# Role of macrophage polarization in heart failure and traditional Chinese medicine treatment

**DOI:** 10.3389/fphar.2024.1434654

**Published:** 2024-07-18

**Authors:** Zheqin Zhu, Min Wang, Shenghua Lu, Sisi Dai, Jianhe Liu

**Affiliations:** ^1^ The First Hospital of Hunan University of Chinese Medicine, Changsha, China; ^2^ Hunan University of Chinese Medicine, Changsha, China

**Keywords:** macrophage polarization, heart failure, traditional Chinese medicine, mechanisms, research progress

## Abstract

Heart failure (HF) has a severe impact on public health development due to high morbidity and mortality and is associated with imbalances in cardiac immunoregulation. Macrophages, a major cell population involved in cardiac immune response and inflammation, are highly heterogeneous and polarized into M1 and M2 types depending on the microenvironment. M1 macrophage releases inflammatory factors and chemokines to activate the immune response and remove harmful substances, while M2 macrophage releases anti-inflammatory factors to inhibit the overactive immune response and promote tissue repair. M1 and M2 restrict each other to maintain cardiac homeostasis. The dynamic balance of M1 and M2 is closely related to the Traditional Chinese Medicine (TCM) yin-yang theory, and the imbalance of yin and yang will result in a pathological state of the organism. Studies have confirmed that TCM produces positive effects on HF by regulating macrophage polarization. This review describes the critical role of macrophage polarization in inflammation, fibrosis, angiogenesis and electrophysiology in the course of HF, as well as the potential mechanism of TCM regulation of macrophage polarization in preventing and treating HF, thereby providing new ideas for clinical treatment and scientific research design of HF.

## 1 Introduction

Heart failure (HF) is the end stage of various heart diseases in which functional and/or structural abnormalities of the heart result in impaired ventricular filling and/or ejection capacity. The main clinical signs and symptoms are caused by stasis in the pulmonary and physical circulation and reduced cardiac output (Bozkurt et al., 2021; Heidenreich et al., 2022; McDonagh et al., 2024). The development of the disease is associated with a synergistic interplay of multiple mechanisms, including dysregulation of the neurohumoral system, oxygen-free radical burst, mitochondrial dysfunction, autophagy, apoptosis, and inflammatory response (Kleinbongard et al., 2010; Dick and Epelman, 2016; Antoine et al., 2017; van der Pol et al., 2019; Yamaguchi, 2019). With high morbidity and mortality rates, HF affects more than 64 million people worldwide and has become a significant public health problem worldwide (Savarese et al., 2023). Drug therapy is the primary treatment for HF, and more and more national HF guidelines recommend quadruple therapy as the primary drug therapy for HF, which can significantly improve the prognosis of patients with HF and open up a “new era” of HF treatment (Vaduganathan et al., 2020; Tromp et al., 2022). However, these medications still have many challenges and limitations, with many adverse effects and recurrent episodes of the disease when taken for an extended period. Traditional Chinese medicine (TCM), with its multi-target and multi-pathway characteristics, has good clinical efficacy in treating HF, especially in regulating the body balance and reducing side effects (Li et al., 2013; Liu et al., 2014).

HF activates the immune system regardless of the factor causing it (Torre-Amione, 2005; Bacmeister et al., 2019). Macrophages are an essential component of the immune system, and activated macrophages produce a variety of cytokines, chemokines, and enzymes that modulate the inflammatory response and promote tissue injury or repair (Murray and Wynn, 2011). After myocardial damage, the local release of chemokines in the tissue promotes the increase of the number of macrophages and differentiation into different phenotypes, thus playing various functions. The characteristics of M1 and M2 macrophage subsets have been well defined. M1 macrophages are pro-inflammatory cells, and M2 macrophages have anti-inflammatory effects (Shapouri-Moghaddam et al., 2018; Zhang H. et al., 2021). Studies have shown that M1 and M2 macrophages can be transformed into each other under different conditions during the course of HF, and the dynamic balance between the two is closely related to tissue damage and repair (Halade et al., 2018; Lv et al., 2020). Therefore, regulating the balance between the two types of macrophages may become essential for treating HF. This article will review the mechanism of macrophage polarization in HF and the research progress of TCM in regulating macrophage polarization for treating HF to provide a new therapeutic strategy.

## 2 Macrophage polarization and mechanisms

### 2.1 Origin and function of macrophages

Macrophages are widely distributed in various body tissues and include monocyte-derived macrophages and tissue-resident macrophages (TRMs) (Moreira Lopes et al., 2020). TRMs are derived from erythroid myeloid progenitors of the yolk sac and fetal monocyte progenitors and are replaced by renewal through *in situ* proliferation when the organism is in a stable state (Epelman et al., 2014; Gentek et al., 2014). When tissue inflammation or injury occurs, TRMs are consumed in large numbers, and monocytes migrate from the periphery to the tissue, acquire the ability to synthesize and secrete inflammatory mediators, and transform into macrophages that play a role in tissue injury (Jakubzick et al., 2017). The main functions of macrophages are pathogen clearance and antigen presentation. TRMs in different tissues have some functional heterogeneity. For example, osteoclasts in bone tissues have bone remodeling effects, and TRMs in the heart play a crucial role in the formation and development of cardiac tissue and blood vessels (Suzuki et al., 2020; Zaman et al., 2021).

### 2.2 Macrophage polarization

Macrophages are highly plastic and differentiate into different subtypes of macrophages with diverse functions under various microenvironments, a process known as macrophage polarization (Yunna et al., 2020). The current paradigm describes two macrophage subpopulations: classically activated M1 macrophages and alternatively activated M2 macrophages (Ruytinx et al., 2018).

#### 2.2.1 Phenotype and function of M1 macrophages

M1 macrophages highly express surface protein markers such as CD16, CD32, CD40, CD68, CD80, CD86, F4/80 h and TRL-4 (Yao et al., 2019; Binatti et al., 2022). Pathogen-associated molecular patterns (PAMPs) such as lipopolysaccharide (LPS), interferon-gamma (INF-γ), tumor necrosis factor-α (TNF-α), and granulocyte-macrophage colony-stimulating factor activate intracellular downstream signaling pathways by binding to different receptors, which promotes M1 macrophage polarization, secretion, and proliferation (Brown et al., 2012). M1 macrophages secrete a large number of pro-inflammatory factors, such as IL-1β, TNF-α, and INF-γ, and chemokines (like chemokine CXC-motif ligand9 (CXCL9), CXCL10, CXCL11, and CXCL16). Simultaneously, immune stress increases reactive oxygen species (ROS) and inducible nitric oxide synthase (iNOS) levels, and the immune response of Th1 and Th17 cells is markedly increased. These responses have pro-inflammatory, anti-tumor, and antigen-presenting effects (Yunna et al., 2020; Tsai et al., 2021; Zhou et al., 2021).

#### 2.2.2 Phenotype and function of M2 macrophages

M2 macrophages are anti-inflammatory and can be polarized into four subtypes: M2a, M2b, M2c and M2d. M2a macrophages are stimulated by IL-4 and IL-13, which express high levels of the CD206 and IL-1Ⅱreceptors. M2a secrete anti-inflammatory substances (such as IL-10, chemokine CC-motif ligand17 (CCL17), CCL18, CCL22, CCL24) and pro-fibrotic factors (TGF-β, insulin-like growth factor (IGF), and fibronectin) reduces chronic inflammation and promotes tissue regeneration and wound healing (Wang et al., 2019). M2b macrophages are mainly induced by immune complexes (ICs) and IL-1β, with high expression of protein markers such as CD86, IL-10R, IL-12R, and tumor necrosis factor superfamily member 14 (TNFSF14). M2b secretes pro-inflammatory factors, including IL-1β, IL-6, and TNF-α, and anti-inflammatory factors like IL-10. Because they have both pro-inflammatory and anti-inflammatory effects, M2b is also called a regulatory macrophage (Mosser and Edwards, 2008). M2c is induced by IL-10, TGF-β and glucocorticoids and is characterized by a high expression of CD163 and CD206 on the cell surface. M2c secretes IL-10 and TGF-β to inhibit inflammation and promote tissue repair, and at the same time, regulates T cells through the secretion of chemokines, such as CCL16 and CCL18, which exert a phagocytic effect on apoptotic cells (Tian L. et al., 2022). M2d, also known as tumor-associated macrophages, can be induced by a combination of TLRs and adenosine receptor agonists or by IL-6 alone and marked by proteins such as IL-10R and IL-12R. M2d secretes IL-10, vascular endothelial growth factor (VEGF), a major inflammatory factor in the tumor environment, promoting angiogenesis and contributing to tumor growth (Wang et al., 2019).

### 2.3 Molecular mechanisms of macrophage polarization

#### 2.3.1 TLRs signaling pathway

Toll-like receptors (TLRs) play a crucial role in regulating macrophage polarization, with TLR4 being the most closely related to M1 macrophages. CD14, a membrane glycoprotein with glycosylated inositol (GPI), enhances the TLR4 signaling pathway, in addition, medullary differentiation protein 2 (MD-2) promotes TLR4 translocation (Park and Lee, 2013). LPS binds to LPS-binding protein (LBP) and recognizes a heterotrimer of CD14/TLR4/MD-2, which activates different downstream signaling pathways such as the myeloid differentiation primary response protein 88 (MyD88) pathway and the TIR structural domain-containing articulators inducing interferon-β (TRIF) signaling pathway (Ciesielska et al., 2021).

MyD88 binds to IL-1 receptor-associated kinase (IRAK) to form a complex (Ciesielska et al., 2021). Subsequently, the complex further interacts with tumor necrosis factor receptor-associated factor 6 (TRAF6), triggering a signaling cascade reaction with TAK1 kinase, which activates inhibitor kappa B kinase α/β (IKK α/β), leading to nuclear translocation of NF-κB transcription factors (Hawiger et al., 1999; Takaesu et al., 2000). The TRIF pathway activated by TLR4 first induces the activation of the ubiquitin ligase TRAF3, followed by the activation of tank-binding kinase 1 (TBK1) and IKKε. After TBK1 phosphorylates the pLxlS consensus motif of TRIF, interferon regulatory factor 3 (IRF3) is recruited. Nuclear translocation occurs after TBK1 phosphorylates IRF3 (Liu et al., 2015). After entering the nucleus, NF-κB and IRF3 bind to target gene binding sites, initiate and regulate the expression of inflammatory factors such as IL-1β and TNF-α, and promote M1 macrophage polarization (Ryu et al., 2022; Mussbacher et al., 2023).

#### 2.3.2 JAK/STAT signaling pathway

Activation of the JAK/STAT signaling pathway regulates macrophage polarization. Ligands bind to corresponding macrophage receptors to form dimers, which recruit and phosphorylate JAK. Activated JAK causes tyrosine phosphorylation of bound receptors to form docking sites for STATs. Subsequently, STATs detach from the receptor and form homologous dimers through SH2 domain-phosphotyrosine interactions. The STATs dimer then undergoes nuclear translocation and binds to relevant target genes to induce macrophage polarization (Ge et al., 2021; Hu et al., 2021). The STAT family consists of seven members, of which STAT1 promotes macrophage polarization to the M1, and STAT3 and STAT6 mediate macrophage conversion to the M2.

The JAK/STAT1 pathway activation by IFN-γ is crucial for M1 macrophage polarization. Unlike IFN-γ, IFN-α/β activated the suppressor of cytokine signaling factor 3 (SOCS3), which prevented STAT1 phosphorylation and M1 polarization (Lawrence and Natoli, 2011). IL-4 activates the JAK/STAT6 pathway, and phosphorylated STAT6 can directly bind to KLF4 and PPAR-γ to mediate M2 polarization by initiating gene transcription (Gong et al., 2017; Chen et al., 2022). In addition, STAT6 increases the expression of the histone demethylase Jmjd3, which demethylates the lysine at position 27 of histone H3 and then acts on the transcription factor interferon regulatory factor 4 (IRF4) to mediate M2 polarization (Ming-Chin Lee et al., 2022). IL-10 is an essential immunosuppressive factor that promotes M2 polarization by activating the JAK/STAT3 pathway (Duncan et al., 2020).

#### 2.3.3 PI3K/Akt signaling pathway

The PI3K/Akt signaling pathway is crucial for macrophage survival, proliferation, migration, and polarization. Signaling factors such as TLR4 and other pathogen recognition receptors, cytokines and chemokines, and Fc receptors activate class I PI3K to convert the second messenger phosphatidylinositol 4,5-diphosphate (PIP2) to phosphatidylinositol 4,5-triphosphate (PIP3) at the plasma membrane. PIP3 attracts Akt and rapamycin complex 2 (mTORC2) and facilitates the activation of Akt by mTORC2 (Vergadi et al., 2017). Activated Akt then activates mTORC1 by inhibiting tuberous sclerosis complex 1/2 (TSC1/2) (Covarrubias et al., 2015).

Macrophages have three distinct Akt subtypes: Akt1, Akt2, and Akt3. It has been observed that Akt1 and Akt2 have opposing effects on regulating macrophage polarization (Arranz et al., 2012). The activation of the PI3K/Akt1 pathway is considered a negative regulator of TLR and NF-kB signaling (Fukao and Koyasu, 2003). On the one hand, the PI3K/Akt1 pathway can upregulate the TLR4 signaling repressor IRAK-M by mediating the inactivation of TRAF6, thereby inhibiting TLR4 target genes and encouraging macrophage polarization toward M2. On the other hand, Akt1 can prevent NF-κB from nuclear transcription, which leads to M2 polarization (Kobayashi et al., 2002; Fan et al., 2010; Tian Y. et al., 2022). Oppositely, Akt2 can facilitate the polarization of macrophages to the M1 by promoting the nuclear translocation of NF-κB, while Akt2 deletion enhances the M2 macrophage phenotype (Babaev et al., 2014; Yin et al., 2023). As a downstream target of PI3K/Akt, mTORC also contributes to macrophage polarization. Studies on how mTORC1 regulates macrophage polarization show contradictory results, possibly due to different environmental activation conditions (Byles et al., 2013; Kimura et al., 2016). In contrast, the mechanism of mTORC2 is relatively straightforward, as it promotes M2 polarization by inducing the transcription factor IRF4 (Huang et al., 2016).

#### 2.3.4 JNK signaling pathway

The JNK signaling pathway is crucial for the polarization and activation of macrophages (Zha et al., 2021). The JNK family contains three isoforms: JNK1, JNK2, and JNK3. Activated JNK controls target gene transcription and expression using downstream substrates such as AP-1 (Koga et al., 2019).

Activation of the JNK signaling pathway is a crucial step in initiating chronic low-grade inflammation (Park and Han, 2024). LPS activates JNK through TLR4/MyD88 signaling and promotes the activation of its downstream substrates, thereby mediating the polarization of M1 macrophages and producing inflammatory factors to promote an inflammatory response (Liu et al., 2018). Furthermore, JNK phosphorylates serine 707 to inactivate STAT6, which causes M1 polarization (Shirakawa et al., 2011). JNK is inhibited by IL-4 and IL-13, leading to macrophage polarization toward M2 and facilitating tumor development, angiogenesis, and metastasis (Cheng et al., 2023).

#### 2.3.5 Notch signaling pathway

Notch receptors are highly conserved throughout evolution, participate in cell growth and development and maintain tissue homeostasis. The mammalian Notch signaling pathway is mainly activated by interactions between three delta-like ligands (DLL1, DLL3, and DLL4), two Jagged family ligands (JAG1 and JAG2), and four transmembrane receptors (Notch1-4) (Eun and Jeong, 2016). Recent studies have shown that the Notch signaling pathway is associated with macrophage activation and polarization (Vieceli Dalla Sega et al., 2019). The precise mechanism is that the intracellular portion (NICD) of Notch is released from the medial membrane into the nucleus following its binding to the ligand, owing to the actions of disintegrin metalloproteinase (ADAM) and gamma-secretase. NICD binds to recombinant signal binding protein-J (RBP-J) to promote M1 polarization. The Notch and TLRs pathways are integrated at the level of IRF8 protein synthesis. Xu et al. showed that the Notch pathway activates TLR4, which induces IRF8 expression and promotes M1 macrophage polarization through the IRAK2-MNK1-elF4E pathway (Xu et al., 2012).

**FIGURE 1:**
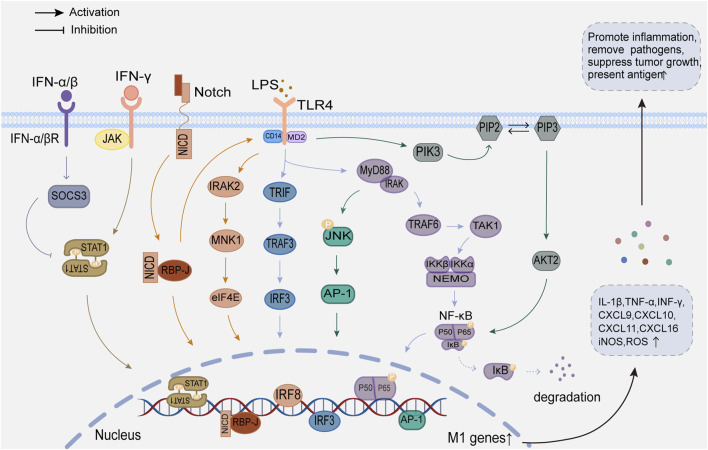
Regulatory mechanisms of M1 macrophage polarization.

**FIGURE 2:**
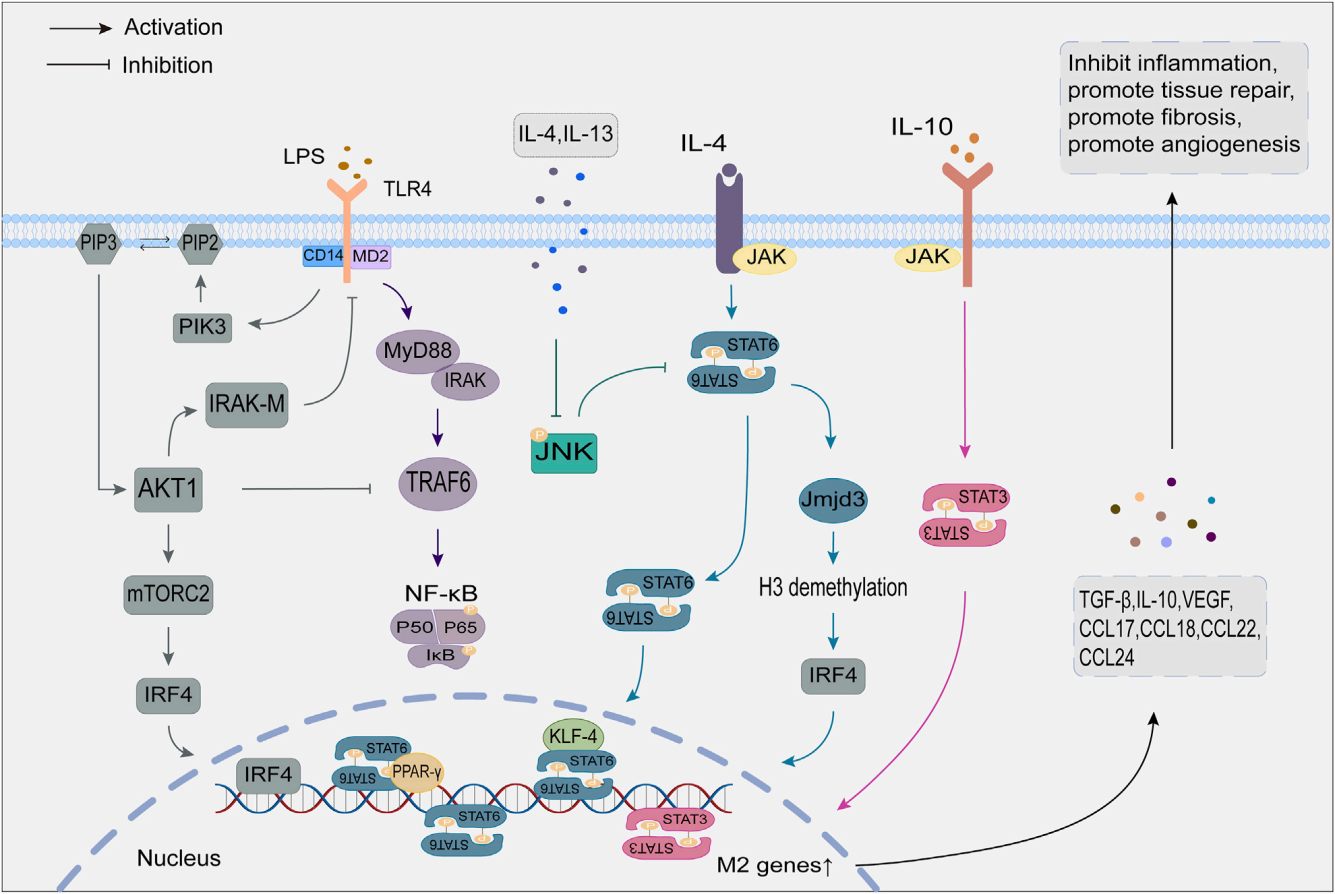
Regulatory mechanisms of M2 macrophage polarization.

The regulatory mechanisms of M1 and M2 macrophage polarization are detailed in Figures 1, 2.

## 3 Role of macrophage polarization in HF

**FIGURE 3:**
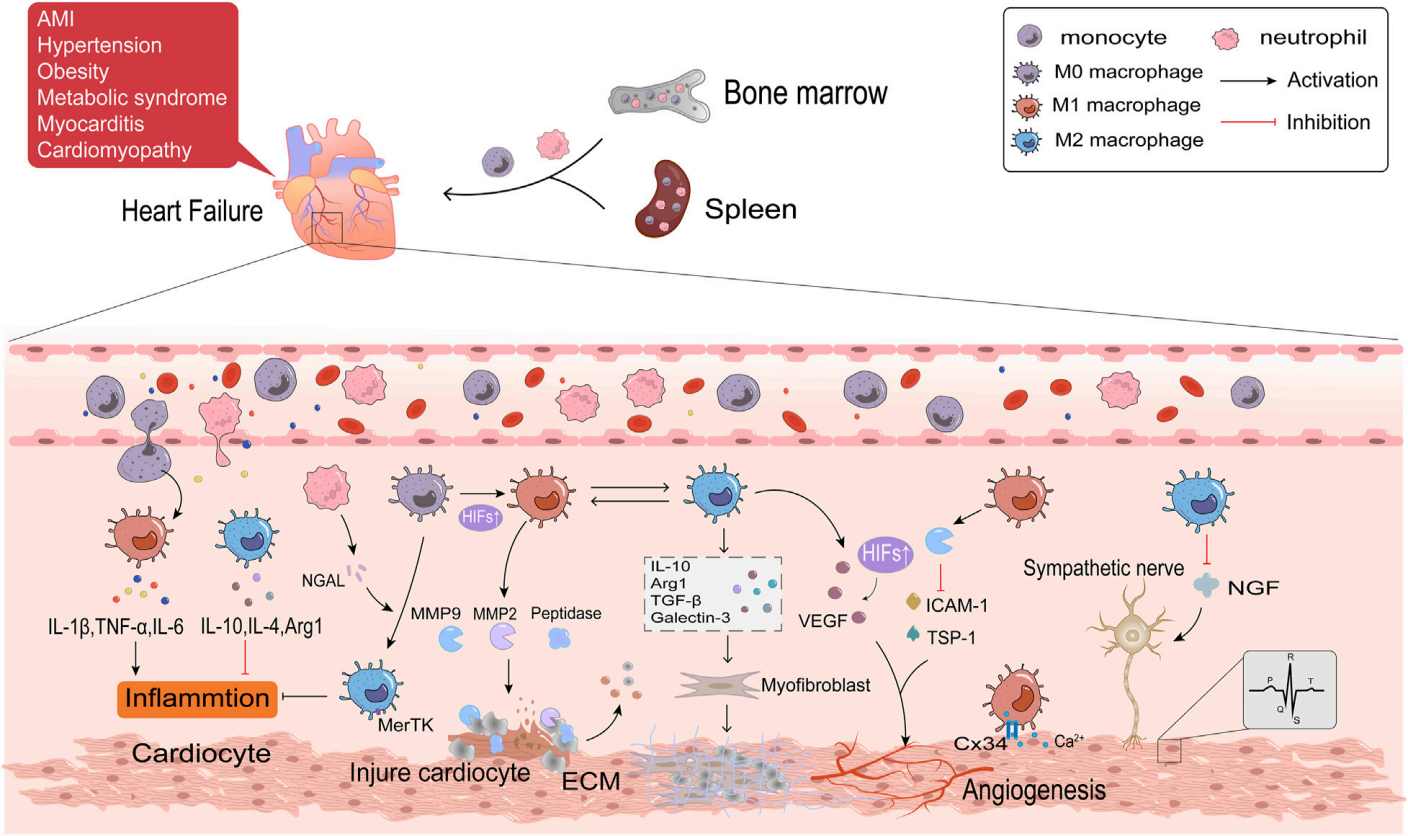
The regulatory mechanisms of macrophage polarization in inflammation, fibrosis, angiogenesis and cardiac electrophysiology of HF.

Multiple complex physiopathologic processes accompany HF. Macrophages are the most numerous immune cells in the heart and are crucial for both preserving cardiac homeostasis and organizing tissue damage healing. After cardiac tissue is damaged, monocytes are recruited from the bone marrow and spleen to the injury site and differentiate into macrophages, which are involved in the various phases of inflammation, repair and remodeling of myocardial injury (Nahrendorf et al., 2007; Swirski et al., 2009). This article will discuss the role of macrophage polarization in HF from the following aspects. The detailed regulatory mechanisms are shown in Figure 3.

### 3.1 Macrophage polarization and inflammation in HF

Acute myocardial infarction (AMI) is the primary cause of HFrEF. Ischemia and subsequent reperfusion injury trigger acute inflammatory responses, which permanently damage cardiomyocytes. Obesity, hypertension, and metabolic syndromes are frequent causes of HFpEF. These diseases can all result in persistent systemic inflammation, which damages cardiomyocytes by producing pro-inflammatory cytokines. Circulating levels of inflammatory mediators have been connected with HF mortality (Mouton et al., 2020). Inflammation also is the primary cause of HF in patients with inflammatory cardiomyopathy or myocarditis (Hanna and Frangogiannis, 2020). This implies that HF is triggered by whatever pathological changes are associated with local and systemic activation of inflammatory signaling cascade responses (Adamo et al., 2020). An initial inflammatory response in HF is triggered by the innate immune response when ischemia and hypoxia cause cardiomyocyte necrosis. This reaction involves the release of danger-associated molecular patterns (DAMPs) such as mitochondrial DNA, activation of complement, and synthesis of inflammatory vesicles (Yang et al., 2015; Rhee and Lavine, 2020). The inflammatory pathway is continuously activated and produces large amounts of inflammatory factors and chemokines, like IL-1, IL-6, TNF-α, and CCL2 (Swirski and Nahrendorf, 2018). Elevated levels of inflammatory factors and chemokines attract neutrophils and monocytes generated in the bone marrow and spleen to travel with the blood to the ischemic myocardium to coordinate the initial inflammatory response and to clear dead cardiomyocytes (Tsou et al., 2007; Zuurbier et al., 2019). When AMI occurs, Ly6Chigh monocytes are the first monocyte population, which differentiate into M1 macrophages, produce protein hydrolases, and secrete matrix metalloproteinases (MMPs) that degrade dead or dying cardiomyocytes and extracellular matrix (ECM) (Nahrendorf et al., 2007; Nahrendorf and Swirski, 2013). The continuous infiltration of immune cells further aggravated oxygen depletion in the infarct area (Van den Bossche et al., 2017). Hypoxia-inducible factors (HIFs) rapidly accumulate in the nucleus of macrophages in response to a hypoxic microenvironment (Zeng et al., 2015). HIFs promotes transcription of the M1 macrophage gene profile and shifts glycolysis to become the primary mode of metabolic energy production in M1 macrophages by driving the expression of the glycolytic gene Slc2al (Fujisaka et al., 2013). Hypoxia stimulates M1 macrophages in the epicardial adipose tissue (EAT) in response to myocardial injury. These macrophages then further penetrate the myocardium and contribute to the exacerbation of cardiac inflammation (Hirata et al., 2011). Researches have indicated that patients with AMI and HFpEF have thicker EAT (Gruzdeva et al., 2018; Horckmans et al., 2018; Gorter et al., 2020). The M2 macrophages predominate in late AMI. In order to support the healing response to AMI, Ly6Clow monocytes are drawn to the myocardium to transform into M2 macrophages (Nahrendorf and Swirski, 2013). Reparative M2c macrophage is also induced by neutrophil gelatinase-associated lipid transport protein (NGAL) (Horckmans et al., 2017). M2c macrophages have a high cytosolic burying capacity and express the Mertk receptor specifically, facilitating tissue healing and inflammation (Wan et al., 2013). Necrotic cardiomyocytes are phagocytosed by macrophages, which provide fatty acids for macrophage mitochondrial respiration and generate NAD + to encourage M2 polarization (Zhang S. et al., 2019). M2 macrophages secrete anti-inflammatory factors such as IL-10, IL-4, and arginase 1 (Arg1), contributing to inflammation regression (Lv et al., 2020). Excessive inflammatory response aggravates a myocardial injury, inhibits tissue repair, promotes adverse LV remodeling, and thus promotes the progression of HF. Therefore, reducing inflammation has become a potent strategy for treating HF (Glezeva et al., 2015). It has been demonstrated that sodium-glucose cotransporter-2 inhibitors (SGLT2i) decrease HF events, particularly HFrEF, perhaps because of their anti-inflammatory properties by lowering plasma glucose and increasing plasma β-hydroxybutyric acid (Bonnet and Scheen, 2018). The β-hydroxybutyric acid promotes the M2 polarization through the STAT6 signaling pathway (Huang et al., 2022).

### 3.2 Macrophage polarization and myocardial fibrosis

Myocardial fibrosis, a common clinical state in many cardiovascular disorders leading to HF, is characterized by the activation of cardiac fibroblasts into myofibroblasts (Lee et al., 2017). Myofibroblasts generate ECM, a crucial factor in developing myocardial fibrosis (Nielsen et al., 2019; Liu Y. et al., 2020). Macrophages may be a key target for slowing the course of HF because of their bidirectional regulatory effect on ECM. M1 macrophages and Ly6Chigh monocytes secrete a variety of proteases, such as MMP2 and MMP9, to clear cell debris and degrade ECM during the acute phase of AMI. Phagocytosis of these cells is a prerequisite for the subsequent replacement of damaged tissue by granulation tissue (Zaidi et al., 2021). As the Warburg effect proceeds, the accumulated lactic acid in the cell causes histone lactation, which raises the M2 gene in macrophages and completes the transition of M1 to M2 macrophages (Zhang D. et al., 2019). Maintaining a balance between reparative and proinflammatory macrophages is crucial since the delayed conversion of M1 to M2 has been identified as one of the primary causes of adverse ventricular remodeling. Macrophages mainly exhibit the M2 phenotype in the repair stage of AMI, which stimulates fibroblasts, enhances ECM protein synthesis, and promotes collagen deposition by producing a multitude of cytokines, such as TGF-β, IL-10, galectin-3, and Arg1 (Hulsmans et al., 2018; Suthahar et al., 2018; Tengbom et al., 2021; Frangogiannis, 2024). TGF-β1 binds to the type II receptor (TGF-βRⅡ) on the surface of fibroblasts, activates the Smad3 signaling pathway, upregulates the expression of smooth muscle actin (α-SMA), and promotes the transformation of fibroblasts into myofibroblasts in the presence of the fibronectin domain EDA (Serini et al., 1998; Kong et al., 2014). In addition to directly activating fibroblasts, galectin-3 modulates immune-inflammatory responses, angiogenesis, and TGF-β amplification of pro-fibrotic signaling by forming lectin-sugar lattices on the cell surface (Chen et al., 2017; Xu GR. et al., 2020; Wang et al., 2023). Arg1 drives L-arginine metabolism to generate ornithine, polyamines, and proline, consequently contributing to fibroblast proliferation and collagen formation (Bhatta et al., 2017). IL-10 mediates the release of osteopontin (OPN) from macrophages through the STAT3-galectin3 pathway, and OPN activates fibroblasts (Shirakawa et al., 2018). Additionally, it has been demonstrated that IL-10 enhances heart function and myocardial wall compliance after myocardial infarction by lowering the collagen I/III ratio (Shirakawa et al., 2018). M2 macrophages produce IL-10 instantaneously in AMI. However, prolonged activation of M2 macrophages under pressure overload ultimately results in increased left ventricular wall thickness, excessive collagen deposition, and progression of diastolic dysfunction (Hulsmans et al., 2018; Zhang S. et al., 2019). M2 macrophages have the ability to differentiate directly into myofibroblasts or fibroblasts, which will accelerate the course of fibrosis. HAIDER et al. evaluated this macrophage-to-fibroblast transformation by examining typical fibroblast markers in MI mice, including type I collagen (COL1A1), prolyl 4-hydroxylase (P4H), fibroblast activating protein (FAP), and α-SMA (Haider et al., 2019). In addition, macrophages participate in angiotensin Ⅱ (AngⅡ) production by synthesizing renin and angiotensin-converting enzymes in stressed myocardium (Ovchinnikov et al., 2020). AngⅡ is an effective stimulant of myocardial fibroblasts. It has been discovered that the salocorticoid receptor inhibits AngⅡ-induced ventricular fibrosis by regulating the polarization of M2 macrophages (Usher et al., 2010). Therefore, the regulation of macrophage polarization will be significant in treating and preventing cardiac fibrosis.

### 3.3 Macrophage polarization and cardiac angiogenesis

When the heart is exposed to stressful stimuli such as pressure overload, ischemia, and hypoxia, compensatory responses are induced to maintain cardiac function, including cardiac hypertrophy and increased angiogenesis. Instead, continuous stimulation can result in maladaptive heart growth, such as fibrosis and thinning of blood vessels, which ultimately leads to the development of HF. Angiogenesis refers to the process of vascular endothelial cells forming new blood vessels after activation, proliferation, and migration under the influence of tissue microenvironment. Macrophages are essential regulators of cardiac angiogenesis. M1 macrophages mainly play an inhibitory role in angiogenesis. In the MI model, M1 macrophages eliminate plenty of exosomes, which highly express pro-inflammatory miRNA, such as miR-155. By suppressing Sirt1/AMPKα2-eNO and RAC1-PAK1/2 signaling pathways, M1 macrophages impair the angiogenesis capacity of endothelial cells, aggravate myocardial injury, and delay cardiac repair (Liu S. et al., 2020). Aging is an independent risk factor for HF. Overexpression of MMP9 derived from M1 macrophages decreases angiogenesis-related factors, such as the expression of intercellular adhesion factor (ICAM-1), platelet/endothelial cell molecule-1 (PECAM-1/CD31), and thrombospondin-1 (TSP1) in the left ventricle. This leads to the exacerbation of cardiac hypertrophy in the case of sparse blood vessels and promotes the progression of cardiac aging (Toba et al., 2017). M2 macrophages, as opposed to M1 macrophages, primarily support the development of new blood vessels. Vascular endothelial growth factor (VEGF), responsible for the rapid growth of collateral blood vessels, is released to compensate for ischemia and induce angiogenesis (Toba et al., 2017). M2 macrophages detect changes in the extracellular matrix environment through integrin α5 (Itga5) during the MI repair phase and activate the cascade of adhesion spot kinase and PI3K to upregulate VEGF-A to promote angiogenesis (Li et al., 2023). VEGF is also activated by HIF-1α, which helps restore blood flow to ischemic tissue (Dai et al., 2007). The proliferation of cardioresident macrophages (M2-like macrophages) in the first week of stress overload is essential for cardiac adaptation and function, and this may be linked to KLF4 by upregulating VEGF-A expression and downregulating anti-angiogenic factors like THBS1. In addition, the infiltration of exogenous macrophages (M1-like macrophages) is detrimental to disease development during the cardiac decompensation period (>4 weeks) in this model, and preventing their infiltration improves myocardial angiogenesis (Liao et al., 2018). Angiogenesis is critical to alleviate myocardial ischemia and hypoxia in HF patients and is an essential issue to be solved urgently in clinical.

### 3.4 Macrophage polarization and cardiac electrophysiology

Arrhythmia is one of the important clinical manifestations of HF, with high morbidity and mortality. The cardiac electrical conduction system is essential for maintaining normal heart rate and function and is a major factor in arrhythmia. Recent studies have demonstrated that macrophages are significant participants in cardiac electrophysiology, acting through various mechanisms, including direct or indirect interactions with other heart cells. Electrical impulses are generated from the SA node and transmitted to the ventricle through the atrioventricular node and conduction pathways. Connexin (Cx) is a significant medium for transmitting electrical signals between cells. Cx in the heart is mainly composed of Cx43, Cx40, Cx45 and Cx30.2, among which Cx43 has the highest content. Cx connects macrophages and cardiac conduction cells to form electrical coupling to regulate the electrophysiological activity of cardiomyocytes (Sugita and Fujiu, 2019). In the MI model, macrophages congregate at the edge of infarct tissue and are polarized into M1 macrophages. Upregulation of potassium channel KCa3.1 in M1 macrophages promotes Ca^2+^ influx to cardiomyocytes *via* Cx43, resulting in prolonged action potential duration APD (Fei et al., 2019). Macrophages can also exert paracrine effects on cardiac electrical activity through various cytokines. M1 macrophages secrete pro-inflammatory factors (TNF-α, IL-1β, and IL-6) to induce cardiac electrical remodeling, in which IL-1β has been shown to reduce the expression of atrial tremor protein (QKI) and further attenuate L-type Ca^2+^ current in cardiocytes to inhibit atrial fibrillation (Tili et al., 2015; Fei et al., 2019). Both the sympathetic and parasympathetic nerves innervate the heart. Arrhythmia is caused by abnormalities in autonomic innervation, and macrophages may be involved in this process (Stavrakis et al., 2020). A study in HF rats demonstrated that depletion of anti-inflammatory macrophages in the stellate ganglion reduces N-type Ca^2+^ currents and excitability of cardiac sympathetic postganglionic (CSP) neurons, thereby reducing cardiac sympathetic overactivation and ventricular arrhythmias in HF (Zhang D. et al., 2021). Nerve growth factor (NGF) is necessary to promote sympathetic nerve germination. M2 macrophages suppress ventricular arrhythmia caused by sympathetic nerve remodeling after myocardial infarction by reducing the expression of NGF (Liu et al., 2023; Yang et al., 2024) found that Sinapic acid (SA) stimulated the PPARγ pathway with concentration-dependent to promote M2 polarization and reduce NGF (Yang et al., 2022). In summary, macrophage polarization is a crucial regulatory factor of cardiac electrical conduction and is expected to be a primary target in the clinical treatment of arrhythmia.

## 4 Clinical dilemmas and treatment strategies for HF

HF can be classified into HFrEF and HFpEF based on the level of left ventricular ejection fraction (LVEF) measured by echocardiography at the time of the patient’s initial evaluation. Angiotensin-converting enzyme inhibitors (ACEI) or angiotensin receptor blockers (ARB)/angiotensin receptor-enkephalin inhibitors (ARNI), beta-blockers, salocorticoid receptor antagonists (MRAs), and SGLT2i constitute guidelines for guided drug therapy (GDMT) for patients with HFrEF. Quadruple therapy has been shown in numerous studies to dramatically lower the risk of death or hospitalization in people with HFrEF (Felker, 2020). Simultaneous initiation of the quadruple therapy and early reach of the target dose are central to the execution of GDMT (Heidenreich et al., 2022). However, a global survey shows that only 25% of patients are likely to start all drugs in GDMT at the same time (Savarese et al., 2024). Drug side effects such as hypotension, creatinine elevation, hyperkalemia, and bradycardia are the main clinical obstacles to the implementation of GDMT. Thus, based on the early intervention concept, it is advised to give preference to small-dose combinations, titrate the dosage, and create a customized regimen in stages based on the patient’s actual situation (Ge et al., 2022). It is important to note that mild hypotension and a slight rise in creatinine do not necessarily require a change in the HF treatment plan (Cautela et al., 2020; Savarese et al., 2024). Hyperkalemia is related to high mortality in HF patients, and part of the reason is that hyperkalemia prompts doctors to discontinue MRAs prematurely (Trevisan et al., 2021). According to researches, patients at risk for hyperkalemia can take advantage of novel potassium binders and the nephroprotective effects of SGLT2i to maintain treatment for MRAs (Pitt et al., 2011; Anker et al., 2015; Ferreira et al., 2021; Butler et al., 2022). Poor patient adherence to multidrug therapy is a non-clinical barrier to GDMT implementation, and outpatient follow-up is currently the most favored approach for this issue (Malmborg et al., 2023). In addition to chronic hypertension, HFpEF has been connected with multiple syndromes and microvascular dysfunction secondary to an enhanced systemic inflammatory state. In terms of the management of HFpEF patients, diuretics are first-line drugs to reduce volume load and blood stasis, and attention is paid to identifying and treating specific causes and the management of complications (McDonagh et al., 2021). Renin-angiotensin-aldosterone system (RAAS) inhibitors and SGLT2i have also demonstrated potential therapeutic advantages in HFpEF in recent years (Solomon et al., 2020; Vaduganathan et al., 2022). Unfortunately, there is no reliable evidence for treatments that improve adverse outcomes in HFpEF patients, which will be a huge challenge to address in future clinical practice. In terms of other treatments, combined with ivabradine, guanylate cyclase agonists, anticoagulants, positive inotropic drugs or TCM, and even cardiac resynchronization (CRT), implantable cardioverter defibrillator (ICD), and other devices are selected according to the individual conditions of patients to reduce the hospitalization rate and mortality of patients with HF.

## 5 The regulatory effect of TCM on macrophages in HF

According to TCM theory, deficiency of qi, blood, yin and yang of the heart, stagnation of qi, blood, and fluid are the pathomechanisms of HF. TCM offers specific benefits in treating HF and has unique advantages in reducing cardiac inflammation, delaying ventricular remodeling, promoting neovascularization, and suppressing arrhythmias (Cao et al., 2021; Dong et al., 2022a; Lu X. et al., 2022; Li et al., 2022). The dynamics of macrophage polarization into M1 and M2 according to the microenvironment can be elaborated by the yin-yang theory of TCM. Yin and yang oppose and restrain one another, just like M1 macrophages exacerbate cardiac inflammation and injury, while M2 macrophages reduce inflammation and repair damaged tissues. The two constrain each other and maintain the balance of yin and yang in the organism to ensure normal physiological functions. Under certain conditions, M1 and M2 transform into each other, called yin-yang transformation in TCM theory. The goal of controlling macrophage polarization is to bring M1 and M2 into balance, consistent with the concept of regulating the balance of yin and yang in TCM. Therefore, TCM regulating macrophage polarization is expected to be one of the most effective ways of treating HF.

### 5.1 TCM compounds alleviate HF by regulating macrophage polarization

According to the pathogenesis of HF in TCM theory, most of the TCM compounds used to treat HF have the effect of warming yang and benefiting qi, promoting blood and fluid circulation. With the deepening of HF research in recent years, more studies have shown that TCM compounds achieve anti-inflammatory, anti-fibrosis, and myocardial tissue protection by regulating the polarization of macrophages. The synergistic effect of complicated components in TCM compounds may explain their various therapeutic effects in HF treatment. Table 1 lists some TCM compounds that regulate the polarization of HF macrophages.

**TABLE 1:** TCM compound alleviates HF by regulating macrophage polarization.

TCM compound	Animal or cellular models	Effect of action	Mechanisms	Refer
Qishen Granule	1)Ligation of the LAD-induced HF in rats and mice 2)LPS induced RAW264.7 cells	1)Inhibited inflammation and myocardial remodeling and promotesd angiogenesis 2)Improved cardiac function	1)AT1-MCP1/CCR2 signaling pathways↓ 2)TGF-β1/Smad3 signaling pathway↓ 3)TLR4-MyD88-NF-κB p65 signaling pathway↓ 4)M1↓,M2↑,AngⅡ↓,MMP2↓,ColⅢ↓,VEGF↑,Ly6C^high^monocytes↓,Ly6C^low^monocytes↑	Lu et al. (2019), Li et al. (2022)
Nuanxinkang	1)Ligation of the LAD-induced HF in mice 2)LPS induced RAW264.7 cells	1)Improved cardiac function 2)Inhibited inflammation, and reduced myocardial fibrosis and apoptosis 3)Inhibited glycolytic capacity	1)IKKβ/IκBα/NF-κB signaling pathway↓; HIF-1α/PDK1 axis↓ 2)IL-1β↓, IL-6↓, TNF-α↓, IL-10↑, M1↓, M2↑(day3), M2↓(day28), basal acidification rate↓, glycolytic reserve capacity↓	Dong et al. (2022a), Dong et al. (2022b)
Shexiang Tongxin Dropping Pill	1)Ligation of the LAD-induced CMD in rats 2)OGD/R-induced human umbilical vein endothelial cells	1)Inhibited vascular lumen stenosis and microvascular leakage, and improved cardiac function 2)Reduced inflammatory cell infiltration and endothelial dysfunction 3)Promoted angiogenesis	1)Dectin-1/Syk/IRF5 signaling pathway↓ 2)PI3K/AKT/mTORC1 signaling pathway ↑ 3)M1↓,M2↑,ZO-1↑,Occludin↑,Claudin-5↑ VE-cadherin↑,iNOS↓,IL-1β↓,IL-10↑,VEGF-A↑	Lu et al. (2022a), Cui et al. (2023)
Naoxintong	Ligation of the LAD induced-HF in mice	1)Improved cardiac function and reduced infarct size 2)Inhibited inflammation	IL-1β↓, P20↓, NLRP3↓, M1↓, M2↑	Wang et al. (2017)
Shenfu Injection	ISO-induced HF in mice	1)Improved cardiac function 2)Inhibited myocardial remodeling	1)TLR4/NF-κB signaling pathway↓ 2)M1↓, M2↑, TNF-α↓, IL-6↓, IL-10↑, Arg-1↑	Yang et al. (2024)
QiShenYiQi Pill	1)Pressure overload-induced cardiac hypertrophy in rats 2)Ang Ⅱ-induced H9C2 cells	1)Improved cardiac function 2)Inhibited myocardial fibrosis and apoptosis	1)RP S19/TGF-β1/Smad signaling pathway↓ 2)M1↓,M2↓,NT-ProBNP↓,caspase-3↓,ATP↑,MMP2↓,MMP9↓,ColⅢ↓	Anwaier et al. (2022)
Xinyin Tablet	TAC-induced HF in mice	1)Improved cardiac function 2)Inhibited myocardial fibrosis and remodeling	1)MLK3/JNK signaling pathway↓ 2)M2↓,TGF-β↓,IL-10↓,Arg-1↓,ColⅠ↓,ColⅢ↓	Liu et al. (2021)
Fangji Fuling Decoction	ISO-induced myocardial fibrosis in mice	1)Inhibited myocardial fibrosis 2)Inhibits inflammation and oxidative stress	M1↓, M2↑, TNF-α↓, IL-1β↓, IL-6↓, IL-10, TGF-β1↓, SOD↓, GSH↓, MDA↑	Shi et al. (2023)

#### 5.1.1 Qishen Granule

Qishen Granule (QSG) is a clinically approved TCM compound for the treatment of HF, which has the effect of warming yang and invigorating qi, promoting blood circulation and detoxifying. QSG consists of *Astragalus mongholicus* Bunge (Fabaceae; *Astragali Radix*), *Salvia miltiorrhiza* Bunge (Lamiaceae; *Salviae miltiorrhizae radix et rhizoma*), *Lonicera japonica* Thunb (Caprifoliaceae; *Lonicerae Japonicae Flos*), *Aconitum carmichaelii* Debeaux (Ranunculaceae; *Aconiti Lateralis Radix Praeparata*), *Scrophularia ningpoensis* Hemsl (Scrophulariaceae; *Scrophulariae Radix*) and *Glycyrrhiza uralensis Fisch* (Fabaceae; *Glycyrrhizae Radix et Rhizoma*). Clinical research has demonstrated that QSG is superior to placebo in decreasing NT-proBNP in CHF patients and enhancing the quality of life, the 6-min walk test (6MWD), the NYHA cardiac function grade, and the symptom score scale (Du et al., 2022). Lu et al. (2019) discovered that QSG could inhibit inflammation and fibrosis and improve the cardiac function of HF rats. Its mechanism was to reduce the recruitment and release of monocytes in damaged myocardial tissues, restrict the conversion of monocytes to M1 macrophages, and promote the M2 macrophage polarization by suppressing the AT1/MCP-1/CCR2 signaling pathway. Decreased M1 macrophages further inhibited TGF-β1/Smad3 pathway-mediated myocardial fibrosis, whereas an increase in M2 macrophages led to a rise in VEGF expression and proliferation in cardiac microvessels to alleviate ischemia and hypoxia in damaged myocardium. Based on previous studies, Li et al. demonstrated *in vitro* that QSG impeded the transformation of Ly6high monocytes to M1 macrophages, possibly by downregulating the expression of the TLR4-MyD88-NF-κB p65 pathway (Li et al., 2022).

#### 5.1.2 Nuanxinkang

Nuanxinkang (NXK) is a simplified TCM compound derived from Xin Yang tablet, which contains *Panax ginseng* C.A.Mey. (Araliaceae; *Ginseng Radix et Rhizoma Rubra*) and *Ilex pubescens* Hook. et Arn (Aquifoliaceae; *Ilex pubescentis radix et caulis*). It has been used for decades in the treatment of HF because it effectively warms yang and invigorates qi, detoxifies, and activates blood circulation (Chen et al., 2019). Dong et al. demonstrated that in MIRI-induced HF mice, NXK reduced the percentage of cardiac M1 macrophage in the acute (days 0–3) and healing phases (days 3–14), lowered levels of blood and cardiac inflammatory cytokines, including TNF-α, IL-1β, and IL-6 to inhibit systemic and local inflammatory, and revealed the mechanism that NXK reduced M1 macrophage by suppressing phosphorylation and nuclear translocation of critical proteins of the IKKβ/IκBα/NF-κB pathway *in vitro*. Additionally, this experiment found that NXK increased M2 macrophages and promoted wound healing in the early but inhibited M2 and the chronic accumulation of TGF-β in the chronic phase (after day 28) to delay collagen fiber aggregation (Dong et al., 2022a). They further explored the regulatory effects of NXK on macrophage energy metabolism and found that NXK depressed the HIF-1α/PDK1 axis to reduce glycolysis in RAW264.7 cells, which ameliorated myocardial injury and slowed down the process of HF (Dong et al., 2022b).

#### 5.1.3 Shexiang Tongxin Dropping Pill

Shexiang Tongxin Dropping Pill (STDP) is composed of *Moschus berezovskii* Flerov (Cervidae; *Moschus*), total ginsenoside of *P. ginseng* C. A. Mey. (Araliaceae; *Ginseng Rhizoma et leafy*), *Bufo gargarizans* Cantor (Bufonidae; *Bufonis Venenum*), *Bos taurus domesticus* Gmelin (Bovidae; *Bovis Calculus*), *Selenarctos thibetanus G.* Cuvier (Ursidae; *Ursi Fellis Pulvis*), *Dryobalanops aromatica* C.F.Gaertn (Dipterocarpaceae; *Borneolum Syntheticum*) and *S. miltiorrhiza* Bunge. As a prescription medication approved by The Chinese State Food and Drug Administration (SFDA), STDP has excellent therapeutic efficacy and has been widely used in the treatment of cardiovascular diseases (Lu et al., 2020; Lin et al., 2022). Clinical studies have shown that compared with trimetazidine alone, STDP combined with trimetazidine to treat ischemic HF can significantly reduce inflammatory factors and neurohormonal factors, restrain left ventricular remodeling, and improve heart function and symptoms of HF (Wu and Hu, 2019). Li et al. induced coronary microvascular dysfunction (CMD) rat and OGD/R-induced endothelial injury model, respectively, proving that STDP alleviates M1 macrophage-related inflammation and endothelial dysfunction by suppressing the Dectin-1/Syk/IRF5 pathway (Cui et al., 2023). According to another research, STDP stimulates M2 macrophages to release VEGF-A, which promotes microangiogenesis, increases blood flow, and improves cardiac function in CMD rats (Lu X. et al., 2022).

#### 5.1.4 Naoxintong

Naoxintong (NXT) originated from the Buyang huanwu decoction in “Correction on the Errors of Mecical Works” by Wang Qingren in the Qing Dynasty. It has a total of 16 herbs and has the effect of promoting blood circulation. A systematic review and meta-analysis of RCT in 1589 CHF patients showed that NXT combined with conventional western medicine was better than conventional Western medicine alone, reduced BNP, and improved cardiac function indicators more effectively (Ban and Ke, 2020). According to reports, NXT protects the heart by encouraging angiogenesis, reducing oxidative stress and inflammation, and controlling the metabolism of fats and carbohydrates (Han et al., 2019). Naoxintong was found to improve heart function and reduce infarct size in MIRI by inhibiting the activation of NLRP3 inflammasome, restricting M1 macrophage polarization and IL-1β to prevent inflammation (Wang et al., 2017).

#### 5.1.5 Shenfu injection

Shenfu injection (SFI) is a modified medication from Shenfu decoction, composed of *P. ginseng* C. A. Mey. (Araliaceae; *Ginseng Radix et Rhizom*a) and *A. carmichaelii* Debeaux can invigorate qi to engender blood and restore yang. SFI is frequently used clinically to treat HF, and a systematic review of RCT involved in 1042 CHF patients revealed that SFI combined with western medicine improved patients’ quality of life and activity tolerance more effectively than using western medicine alone (Zhou et al., 2023). Another similar systematic review and meta-analysis of RCT in 3231 HF patients showed that SFI adjuvant therapy was superior to conventional western medicine alone in terms of clinical efficacy and improved cardiac function indicators and could reduce the incidence of adverse reactions (Wu et al., 2022; Yang et al., 2024). Established the HF mice by intraperitoneal injection of isoproterenol (ISO) and observed the M1/M2 imbalance was mainly caused by the increase of M1 macrophages. SFI can inhibit M1 macrophage activation, significantly reduce TNF-α and IL-6 secretion, promote macrophage polarization towards M2, release Arg1 and IL-10, reduce inflammation, and improve cardiac function and ventricular remodeling by downregulating the TLR4/NF-κB signaling pathway (Yang et al., 2024).

#### 5.1.6 QiShenYiQi pill

QiShenYiQi Pill (QSYQ) is a compound approved by the SFDA that benefits qi and promotes blood circulation. It is composed of *A. mongholicus* Bunge, *S. miltiorrhiza* Bunge, and *Panax notoginseng* (Burk.)F. H. Chen (Araliaceae; *Notoginseng Radix*), and *Dalbergia odorifera* T. Chen (Leguminosae; *Dalbergiae Odoriferae Lignum*). Numerous clinical investigations have shown that QSYQ is an effective treatment for HF. A systematic review and meta-analysis involving 895 patients with HFpEF revealed that western medicine combined with QSYQ could better decrease BNP and increase the rate of cardiac function improvement and 6-MWD compared with the western medicine group (Wang et al., 2021). Research has demonstrated that high-dose QSYQ (0.8 g/kg) reduces cardiac hypertrophy induced by pressure overload, restrains cardiomyocyte apoptosis, and the expression of our-and-a-half LIM domains protein 2 (FHL2), inhibits M1/M2 macrophage polarization and myocardial fibrosis mediated by RP S19/TGF-β1/Smad signaling pathway (Anwaier et al., 2022).

#### 5.1.7 Xinyin Tablet

Xinyin Tablet (XYT) comprises *P. ginseng* C. A. Mey., *A. mongholicus* Bunge, *Schisandra Chinensis* (Turcz.)Baill. (Magnoliaceae; S*chisandrae Chinensis Fructus*), *Leonurus japonicus* Houtt. (Lamiaceae; *Leonuri Herba*), *Descurainia sophia* (L.). Webb ex Prantl (Brassicaceae; *Descurainiae Semen*), and *I. pubescens* Hook. et Arn. It is a hospital preparation approved by the Guangdong Food and Drug Administration (approval number: Yueyao Z20071178) and has been in clinical use for HF for several decades (Liu Q. et al., 2020). Clinical research has demonstrated that the combination of XYT can considerably improve the left ventricular systolic and cardiopulmonary function in male CHF patients when used by standardized western medicine treatment (Ye et al., 2019). The myocardial tissue of CHF mice resulting from transverse aortic constriction (TAC) exhibited significant collagen fiber deposition, a marked degree of myocardial fibrosis, and elevated expression of M2 macrophage markers. Through restricting the MLK3/JNK signaling pathway, XYT inhibits the transformation of macrophages into M2 and the release of anti-inflammatory factors, thereby reducing the synthesis of Col I and Col Ⅲ in myocardial tissue and abnormal deposition in the extracellular stroma, alleviating myocardial fibrosis and delaying ventricular remodeling (Liu et al., 2021).

#### 5.1.8 Fangji Fuling Decoction

Fangji Fuling Decoction (FFD), derived from “Synopsis of Golden Chamber” by Zhang Zhongjing in the Han Dynasty, is composed of five herbs: *Stephania tetrandra* S.Moore (Menispermaceae; *Stephaniae Tetrandrae Radix*), *Poria cocos* (Schw.) Wolf (Polyporaceae; *Poria*), *Cinnamomum verum* J.Presl (Lauraceae; *Cinnamomi Ramulus*), *A. mongholicus* Bunge and *G. uralensis* Fisch. It has the effect of warming yang, invigorating qi and promoting blood and fluid circulation, and is a frequently used compound in clinical treatment of HF (Wang and Lu, 2015). According to studies, FFD inhibit inflammation, decrease collagen deposition in mice with myocardial fibrosis, increase the expression of the anti-inflammatory factor IL-10, decrease the expression of pro-inflammatory factors TNF-α, IL-1β and IL-6, and promote the transformation of macrophage from M1 to M2 (Shi et al., 2023).

### 5.2 Active ingredient of single TCM alleviate HF by regulating macrophage polarization

Through the analysis of the rule of medicine in the treatment of HF by TCM, it is concluded that the main effect of medicine for HF is to warm yang and benefit qi, promote blood circulation and remove blood stasis. The active ingredients of single TCM are the material basis for its effect. The studies demonstrated how TCM active ingredients modulate M1/M2 macrophage polarization balance in various ways, potentially aiding in the treatment of HF. Table 2 lists some active ingredients of single TCM that regulate the polarization of HF macrophages.

**TABLE 2:** TCM active ingredient alleviates HF by regulating macrophage polarization.

TCM active ingredient	Source	Animal or cellular models	Effect of action	Mechanisms	Refer
Puerarin + Tanshinone IIA	*Pueraria lobata* (Willd.) Ohwi + *Salvia miltiorrhiza* Bunge	1)Ligation of the LAD-induced MI in mice 2)LPS-induced RAW264.7 cells	1)Improved cardiac function and hemodynamics 2)Inhibited inflammation 3)Attenuated cardiac fibrosis	LDH↓,CK↓,CK-MB↓,M1↓,M2↑,IL-6↓,IL-1β↓,iNOS↓,IL-10↑,α-SMA↓, TLR4↓, C/EBP-β↑	Gao et al. (2019)
Dihydrotanshinone I	*Salvia miltiorrhiza* Bunge	1)DOX induced-DIC in zebrafish and mice 2)LPS-induced RAW264.7 cells and DOX-induced H9C2 cell	1)Improved cardiac function 2)Inhibited inflammation	1)TFEB-IKK-NF-κB inflammatory signaling axis↓ 2)M1↓,TNF-α↓,IL-1β↓,p-NF-κB↓,COX2↓,IL-8↓,p-mTOR↓	Wang et al. (2020)
Salvianolic acid B	*Salvia miltiorrhiza* Bunge	Ligation of the LAD-induced MI/R in mice	1)Inhibited inflammation and glycolysis 2)Improved cardiac function 3)Preserved cardiac morphology and structure	M1↓,M2↑,TNF-α↓,IL-6↓,IL-1β↓,Arg1↑,Clec10a↑,Mrc↑,mTORC1, ECAR↓, lactate↓	Zhao et al. (2020)
Curcumin	*Curcuma longa* L	1)Cardiac myosin-induced EAM in rats 2)IL-4 and IL-13 induced-RAW264.7 cells 3)Ligation of the LAD-induced MI in mice 4)M-CSF induced-BMM.	1)Ameliorated heart injury 2)Inhibited inflammation 3)Reduced infarct size and myocardial fibrosis 4)Improved cardiac function	M1↓,M2↑,IL-4↑,IL-13↑,STAT6↑,MMR↑,Arg1↑,IL-1β↓,iNOS↓,TNF-α↓,IL-1β,IL-6↓,IL-10↑,AMPK↓	Gao et al. (2015), Yan et al. (2021)
Latifolin	*Dalbergia odorifera* T. Chen	1)DOX-induced DIC in mice 2)Peritoneal Macrophage in mice	1)Improved cardiac function 2)Inhibited inflammation	LDH↓,M1↓,M2↑,iNOS↓,CD86↓,CD206↑,IL-10↑,IL-4R↑,TNF-α↓,IL-1β↓,IL-6↓	Zhang et al. (2020a)
Arctigenin	*Arctium lappa* L	1)Ligation of the LAD-induced MI in mice 2)LPS induced-RAW264.7 cells	1)Alleviated postinfarction cardiac injury 2)Inhibited inflammation	TNF-α↓,IL6↓,M1↓,M2c↓,M2a↑,M2b↑,M2d↑,NFAT5↓,p-JAK2↓,p-STAT1↓,p-IKBα↓,p-P65↓	Ni et al. (2020)

#### 5.2.1 Puerarin and tanshinone ⅡA

Puerarin (Pue), the primary active ingredient of *Pueraria lobata* (Willd.) Ohwi (Leguminosae; *Puerariae Lobatae Radix*) has a wide range of pharmacological effects and is closely related to cardiovascular diseases. Studies have shown that Pue inhibits cardiac hypertrophy by reducing the generation of ROS and inhibiting the activation of ERK1/2, p38 MAPK and NF-κB pathways (Chen et al., 2014). Tanshinone ⅡA is one of the active ingredients of *S. miltiorrhiza* Bunge, which has therapeutic value in alleviating cardiac oxidative stress, inflammation and fibrosis (Lu TC. et al., 2022). The combination of tanshinone ⅡA and Pue in treating ischemic heart disease has a synergistic effect. The two combined at a 1:1 ratio for 28 days have been shown in an experiment to significantly improve cardiac dysfunction resulting from AMI. This improvement may be attributed to the inhibition of M1 macrophage and the promotion of M2 macrophage during the early stages of inflammation, reducing collagen synthesis and inhibiting myocardial fibrosis and ventricular remodeling (Gao et al., 2019).

#### 5.2.2 Dihydrotanshinone Ⅰ

Dihydrotanshinone I (DHT) is another active ingredient of *S. miltiorrhiza* Bunge, which has a protective effect on myocardial injury. DHT significantly reduces the oxidative stress damage of H9C2 cells induced by OGD/R to reduce cell apoptosis (Wang et al., 2024). Doxorubicin (DOX) -induced cardiac inflammation is a high-risk factor for HF. Wang et al. discovered that DHT improves DOX-induced cardiac dysfunction by regulating the mTOR-TFEB-NF-κB signaling pathway, reducing M1 macrophage polarization and the release of TNF-α and IL-1β, and controlling inflammation (Wang et al., 2020).

#### 5.2.3 Salvianolic acid B

Salvianolic acid B (Sal B) is also one of the active components of *S. miltiorrhiza* Bunge, which has anti-oxidation, anti-MIRI, and anti-atherosclerosis effects and has positive impacts on cardia-cerebrovascular disease (He et al., 2023). It has been reported that in MIRI mice, Sal B reduces M1 macrophages and increases M2 macrophages after reperfusion for 3 days by inhibiting mTORC1-induced glycolysis, thus reducing collagen deposition, improving cardiac dysfunction, and restricting inflammation following MIRI (Zhao et al., 2020).

#### 5.2.4 Curcumin

Curcumin, the primary active ingredient of *Curcuma longa* L. (Zingiberaceae; *Curcumae Longae Rhizoma*), is a regulator of macrophage polarization, which plays a cardioprotective role through various signaling pathways (Zhou et al., 2015). Studies have found that curcumin activates the STAT6 pathway through the secretion of IL-4 and IL-13, promotes the polarization of M2 macrophages, reduces the infiltration of proinflammatory cells, and enhances the cardiac function indicators of dilated cardiomyopathy induced by autoimmune myocarditis (Gao et al., 2015). Subsequently, Yan et al. investigated how curcumin affected macrophage polarization in MI mice, and the results showed that curcumin decreased M1 macrophages, TNF-α, IL-1β and IL-6 through the AMPK pathway, increased M2 macrophages and IL-10, inhibited early inflammation, and thus impaired myocardial remodeling after 3 months of MI (Yan et al., 2021).

#### 5.2.5 Latifolin

*Dalbergia odorifera* T. Chen is an herb that promotes blood circulation and relieves pain. Latifolin is one of the active ingredients of *D. odorifera* T. Chen. According to pharmacological research, Latifolin possesses antithrombotic, anti-inflammatory, and antioxidant properties (Yang et al., 2013). Latifolin suppressed the expression of M1 biomarkers (iNOS, CD86), enhanced the expression of M2 biomarkers (CD206, IL-10, IL-4R), and decreased the secretion of TNF-α, IL-1β, and IL-6 to block cardiac inflammation caused by DOX plays a role in cardiac protection (Zhang N. et al., 2020).

#### 5.2.6 Arctigenin

Arctigenin (ATG) is the essential ingredient in *Arctium lappa* L. (Asteraceae, *Arctii Fructus*), possessing anti-oxidative stress, anti-cancer and anti-inflammatory properties (Gu et al., 2012; Xu X. et al., 2020; Guo et al., 2020). Ni et al. revealed that ATG reduces cardiac inflammation, enhances cardiac function, shrinks infarct size, prevents M1/M2c macrophage polarization, and promotes M2a/M2b/M2d polarization in MI mice. Utilizing RNA-Seq analysis, they proceeded to ascertain the regulatory mechanism of ATG on macrophages. Their findings were validated in subsequent *in vitro* and *in vivo* experiments, which indicated that ATG regulation of macrophage polarization was associated with inhibition of the JAK-STAT and NF-κb pathways induced by NFAT5 genes (Ni et al., 2020).

### 5.3 Acupuncture alleviates HF by regulating macrophage polarization

Acupuncture, a common complementary and integrative therapy, has been used clinically by millions worldwide. It specifically improves angina pectoris, palpitations, and other systemic diseases. Anti-inflammation is one of the therapeutic mechanisms of acupuncture related to regulating macrophage polarization. In AMI mice induced by LAD ligation, electroacupuncture pretreatment at Neiguan (PC6) for 3 days inhibited the activation of the NLRP3 inflammasome, promoted M2 macrophages polarization, and reduced the degree of inflammation after AMI injury, thereby reducing infarct size and improving cardiac function (Zhang T. et al., 2020). Hua et al. detected and sampled MIRI rats at 6 h, 24 h, and 3 days after reperfusion, which further proved that electroacupuncture accelerated the M2 macrophages polarization, promoted the transition from the acute proinflammatory phase to the anti-inflammatory repair phase after MIRI, and ultimately produced cardiac protection (Bai et al., 2023).

## 6 Conclusion and prospects

With the prevalence of coronary heart disease, hypertension, obesity, diabetes, and other diseases, the morbidity and mortality of HF are increasing annually, which seriously affects people’s lives and social development. At present, there are still many barriers in the treatment of HF, so it is urgent to develop new therapeutic strategies.

In recent years, the theory of immune inflammation has gained widespread attention. Macrophages are a crucial component of the immune system that is capable of rapidly detecting and reacting to environmental changes, regulating inflammation, and repairing tissues by polarizing into M1 or M2 macrophages. During the course of HF, macrophages sense early cardiac injury through various signaling molecules. M1 macrophages secrete pro-inflammatory factors mediating early inflammation, removing pathogens and damaged myocardium to exert a cardioprotective effect. However, excessive or persistent inflammation results in massive death of cardiomyocytes and aggravates cardiac injury. As the disease progresses, M2 macrophages release anti-inflammatory factors to suppress the inflammation, promote fibroblast activation and collagen deposition, and repair damaged heart, but immoderate fibrosis causes cardiac stiffness and deterioration of cardiac function. The regenerative capacity of microvessels and the electrophysiological activity of cardiomyocytes are critical to cardiac function and closely related to macrophage polarization. It follows that maintaining a balance of M1/M2 macrophages is a potential target for treating HF.

TCM has a broad clinical foundation for treating and preventing HF, and long-term practice has shown its safety and effectiveness. The function of M1 and M2 macrophages is mutually restricted, consistent with TCM yin-yang theory. The pathogenesis of HF is usually the decline of heart yang qi and the stagnation of blood and fluid. Therefore, TCM, which regulates the polarization of macrophages, has the effect of warming yang, invigorating qi, and promoting blood and fluid circulation. Although TCM has become increasingly aware of the function that macrophage polarization plays in HF, there are few studies on TCM compound regulation of macrophage polarization. In addition, existing studies mainly focus on inflammation and are limited to detecting M1/M2 macrophage surface protein markers and inflammatory factors secreted by macrophages. Some studies also ignored the regulatory pathway mechanism, which leads to an inadequate integration of evidence chains. It is unable to meet the methodical understanding of TCM treatment for HF. Furthermore, most researches are restricted to animal and cellular levels and lack clinical evidence. TCM has the characteristics of multi-component, multi-target and multi-link action, and has varying functions in different stages of disease development. In the future, it is necessary to strengthen further the basic and clinical research of TCM intervention in HF, especially TCM compounds. Meanwhile, the mechanism of TCM regulating macrophage polarization in different pathological stages of HF will be explored to enrich the scientific connotation of TCM treatment of HF and highlight the scientific and effectiveness of TCM yin-yang theory.
