# Supplementary material for: Role of macrophage polarization in heart failure and traditional Chinese medicine treatment
Source: Front Pharmacol. 2024 Jul 18;15:1434654. doi: 10.3389/fphar.2024.1434654 (PMC11298811; doi:10.3389/fphar.2024.1434654)
Supplement: Supplementary file 1 [file DataSheet1.ZIP › Supplementary files/Table2.docx]

| **Table 2 TCM active ingredient alleviates HF by regulating macrophage polarization.** | | | | | |
| --- | --- | --- | --- | --- | --- |
| **TCM**  **active ingredient** | **Source** | **Animal or cellular models** | **Effect of action** | **Mechanisms** | **Refer** |
| Puerarin  +  Tanshinone IIA | *Pueraria lobata* (Willd.) Ohwi +*Salvia miltiorrhiza* Bunge | 1)Ligation of the LAD-induced MI in mice.  2)LPS-induced RAW264.7 cells | 1)Improved cardiac function and hemodynamics  2)Inhibited inflammation  3)Attenuated cardiac fibrosis | LDH↓,CK↓,CK-MB↓,M1↓,M2↑,IL-6↓,IL-1β↓,iNOS↓,IL-10↑,α-SMA↓, TLR4↓, C/EBP-β↑ | [156] |
| Dihydrotanshinone I | *Salvia miltiorrhiza* Bunge | 1)DOX induced DIC in zebrafish and mice.  2)LPS-induced RAW264.7 cells and DOX-induced H9C2 cell. | 1)Improved cardiac function.  2)Inhibited inflammation. | 1)TFEB-IKK-NF-κB inflammatory signalling axis↓  2)M1↓,TNF-α↓,IL-1β↓,p-NF-κB↓,COX2↓,IL-8↓,p-mTOR↓ | [161] |
| Salvianolic acid B | *Salvia miltiorrhiza* Bunge | Ligation of the LAD -induced MI /R in mice. | 1)Inhibited inflammation and glycolysis.  2)Improved cardiac function.  3)Preserved cardiac morphology and structure. | M1↓,M2↑,TNF-α↓,IL-6↓,IL-1β↓,Arg1↑,Clec10a↑,Mrc↑,mTORC1, ECAR↓, lactate↓ | [163] |
| Curcumin | *Curcuma longa* L. | 1)Cardiac myosin-induced EAM in rats.  2)IL-4 and IL-13 induced RAW264.7 cells.  3)Ligation of the LAD-induced MI in mice.  4)M-CSF induced BMM. | 1)Ameliorated heart injury.  2)Inhibited inflammation.  3)Reduced infarct size and myocardial fibrosis.  4)Improved cardiac function. | M1↓,M2↑,IL-4↑,IL-13↑,STAT6↑,MMR↑,Arg1↑,IL-1β↓,iNOS↓,TNF-α↓,IL-1β,IL-6↓,IL-10↑,AMPK↓ | [166-167] |
| Latifolin | *Dalbergia odorifera* T. Chen | 1)DOX-induced DIC in mice.  2)Peritoneal Macrophage in mice. | 1)Improved cardiac function.  2)Inhibited inflammation. | LDH↓,M1↓,M2↑,iNOS↓,CD86↓,CD206↑,IL-10↑,IL-4R↑ ,TNF-α↓,IL-1β↓,IL-6↓ | [169] |
| Arctigenin | *Arctium lappa* L. | 1)Ligation of the LAD induced MI in mice .  2)LPS induced RAW264.7 cells. | 1)Alleviated postinfarction cardiac injury.  2)Inhibited inflammation. | TNF-α↓,IL6↓,M1↓,M2c↓,M2a↑,M2b↑,M2d↑,NFAT5↓,p-JAK2↓,p-STAT1↓,p-IKBα↓,p-P65↓ | [173] |
