# Supplementary figures and images for: Role of macrophage polarization in heart failure and traditional Chinese medicine treatment
Source: Front Pharmacol. 2024 Jul 18;15:1434654. doi: 10.3389/fphar.2024.1434654 (PMC11298811; doi:10.3389/fphar.2024.1434654)

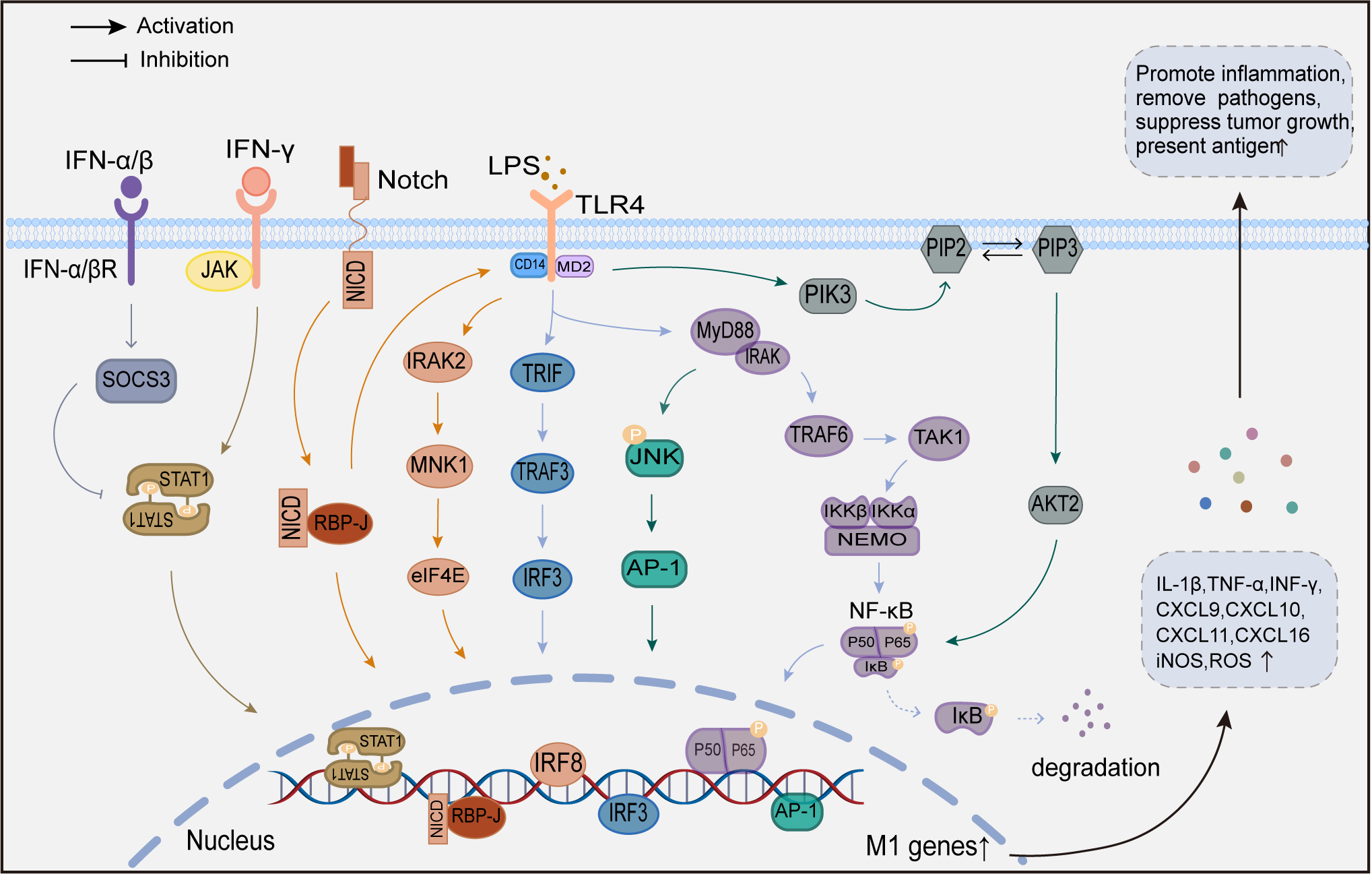

Supplement: Supplementary file 1 [file DataSheet1.ZIP › Supplementary files/Regulatory mechanisms of M1 macrophage polarization..tif]

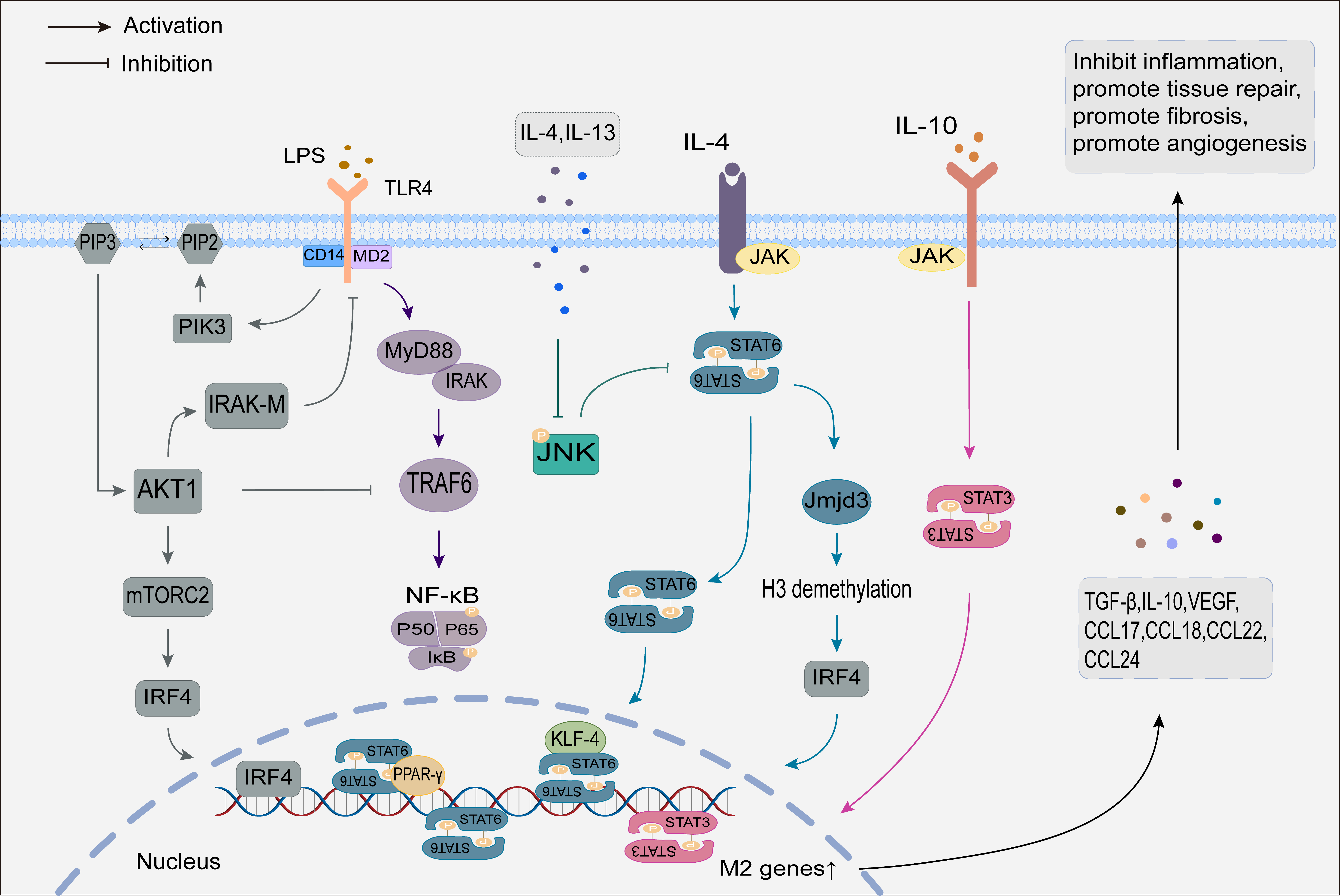

Supplement: Supplementary file 1 [file DataSheet1.ZIP › Supplementary files/Regulatory mechanisms of M2 macrophage polarization..tif]

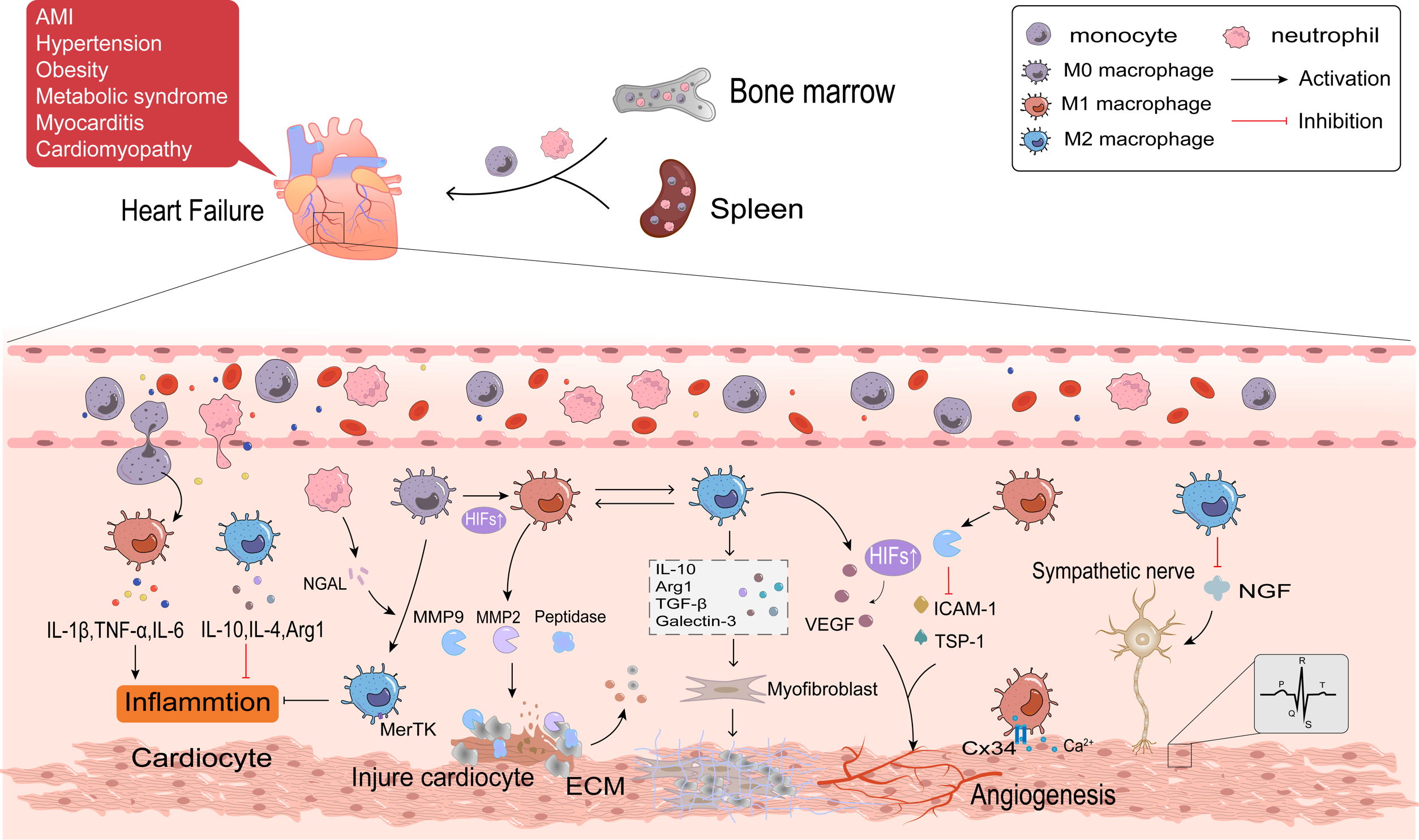

Supplement: Supplementary file 1 [file DataSheet1.ZIP › Supplementary files/The regulatory mechanism of macrophage polarization in inflammation, fibrosis, angiogenesis and cardiac electrophysiology of HF..tif]
