# Supplementary material for: Role of macrophage polarization in heart failure and traditional Chinese medicine treatment
Source: Front Pharmacol. 2024 Jul 18;15:1434654. doi: 10.3389/fphar.2024.1434654 (PMC11298811; doi:10.3389/fphar.2024.1434654)
Supplement: Supplementary file 1 [file DataSheet1.ZIP › Supplementary files/Table 1.doc]

| **Table 1 TCM compound alleviates CHF by regulating macrophage polarization.** | | | | |
| --- | --- | --- | --- | --- |
| **TCM**  **compound** | **Animal or cellular models** | **Effect of action** | **Mechanisms** | **Refer** |
| Qishen Granule | 1)Ligation of the LAD-induced HF in rats and mice.  2)LPS induced RAW264.7 cells. | 1)Inhibited inflammation and myocardial remodeling and promotesd angiogenesis.  2)Improved cardiac function. | 1)AT1-MCP1/CCR2 signaling pathways↓  2)TGF-β1/Smad3 signaling pathway↓ 3)TLR4-MyD88-NF-κB p65 signalling pathway↓  4)M1↓,M2↑,AngⅡ↓,MMP2↓,ColⅢ↓,VEGF↑,Ly6C^high^monocytes↓,Ly6C^low^monocytes↑ | [146,141] |
| Nuanxinkang | 1)Ligation of the LAD induced HF in mice.  2)LPS induced RAW264.7 cells. | 1)Improved cardiac function.  2)Inhibited inflammation ,and reduced myocardial fibrosis and apoptosis.  3)Inhibited glycolytic capacity. | 1)IKKβ/ IκBα/ NF-κB signaling pathway↓; HIF-1α/PDK1 axis↓.  2)IL-1β↓，IL-6↓，TNF-α↓，IL-10↑，M1↓，M2↑（day3），M2↓（day28），basal acidification rate↓，glycolytic reserve capacity↓ | [143,148] |
| Shexiang Tongxin Dropping Pill | 1)Ligation of the LAD induced CMD in rats.  2)OGD/R-inducedHuman umbilical vein endothelial cells. | 1)Inhibited vascular lumen stenosis and microvascular leakage, and improved cardiac function.  2)Reduced inflammatory cell infiltration and endothelial dysfunction.  3)Promoted angiogenesis. | 1)Dectin-1/Syk/IRF5 signaling pathway↓  2)PI3K/AKT/mTORC1 signaling pathway ↑  3)M1↓,M2↑,ZO-1↑,Occludin↑,Claudin-5↑,  VE-cadherin↑,iNOS↓,IL-1β↓,IL-10↑,VEGF-A↑ | [142,152] |
| Naoxintong | Ligation of the LAD induced HF in mice. | 1)Improved cardiac function and reduced infarct size.  2)Inhibited inflammation. | IL-1β↓，P20↓，NLRP3↓，M1↓，M2↑ | [155] |
| Shenfu Injection | ISO-induced HF in mice. | 1)Improved cardiac function.  2)Inhibited myocardial remodeling | 1)TLR4/NF-κB signaling pathway↓  2）M1↓，M2↑，TNF-*α*↓，IL-6↓，IL-10↑，Arg-1↑ | [158] |
| QiShenYiQi Pill | 1) Pressure overload-induced cardiac hypertrophy in rats. 2) Ang Ⅱ-induced H9C2 cells. | 1)Improved cardiac function.  2)Inhibited myocardial fibrosis and apoptosis. | 1)RP S19/TGF-β1/Smad signaling pathway↓  2)M1↓,M2↓,NT-ProBNP↓,caspase-3↓,ATP↑,MMP2↓,MMP9↓,ColⅢ↓ | [160] |
| Xinyin Tablet | TAC-induced HF in mice. | 1)Improved cardiac function  2)Inhibited myocardial fibrosis and remodeling | 1)MLK3/JNK signaling pathway↓  2)M2↓,TGF-β↓,IL-10↓,Arg-1↓,ColⅠ↓,ColⅢ↓ | [163] |
| Fangji Fuling Decoction | ISO-induced myocardial fibrosis in mice. | 1)Inhibited myocardial fibrosis.  2)Inhibits inflammation and oxidative stress | M1↓, M2↑, TNF-α↓, IL-1β↓ , IL-6↓, IL-10, TGF-β1↓, SOD↓, GSH↓, MDA↑ | [165] |
